# Inconclusive prospective associations between food insecurity, self-esteem, and depressive symptoms: a comment on Baek and Yoon (2026)

**DOI:** 10.3389/fpsyg.2026.1866873

**Published:** 2026-07-31

**Authors:** Kimmo Sorjonen, Bo Melin, Marika Melin

**Affiliations:** Department of Clinical Neuroscience, Karolinska Institutet, Stockholm, Sweden

**Keywords:** depressive symptoms, food insecurity, juxtaposition of findings, random-intercept cross-lagged panel model (RI-CLPM), self-esteem

## Abstract

Based on findings with the random-intercept cross-lagged panel model (RI-CLPM), Baek and Yoon concluded that food insecurity and poor psychological health exacerbate each other in a vicious cycle. However, the RI-CLPM is susceptible to spurious findings. Here, we analyzed data with alternative models and found divergent decreasing and increasing prospective effects between food insecurity and self-esteem and depressive symptoms. Hence, the findings by Baek and Yoon may have been spurious and their conclusions premature. It is important for researchers to bear in mind that correlations, including effects in the RI-CLPM, in observational (i.e., non-experimental) data may be spurious. We recommend researchers to fit alternative models to data and to base conclusions on a juxtaposition of findings.

## The random-intercept cross-lagged panel model (RI-CLPM)

The random-intercept cross-lagged panel model (RI-CLPM) is an extension of the traditional cross-lagged panel model (CLPM). In the RI-CLPM, autoregressive and cross-lagged effects between two (or more) constructs are estimated while adjusting for individuals’ stable trait-like levels of the constructs. This way, effects are estimated within individuals rather than between individuals as in the traditional CLPM (Hamaker et al., 2015; Mulder and Hamaker, 2021). Within-individual effects are often assumed to be less biased and, hence, better estimates of true prospective effects compared with between-individual effects (Hamaker et al., 2015; Usami et al., 2019). However, the RI-CLPM cannot adjust for time-varying confounders (Murayama and Gfrörer, 2025; Rohrer and Murayama, 2023). This means that the RI-CLPM is susceptible to similar spurious findings as the traditional CLPM (Sorjonen and Melin, 2025b).

## The study by Baek and Yoon

We refer to Baek and Yoon (2026) for more comprehensive information on the study sample, used instruments, procedures, etc. In short, they analyzed data on self-rated food insecurity, self-esteem, and depressive symptoms with the RI-CLPM. Data was collected from 13,893 Korean adults (56.7% women, mean age at baseline = 53.3 years) annually between 2012 and 2019. Based on statistically significant negative within-individual cross-lagged effects between food insecurity and self-esteem, and positive effects between food insecurity and depressive symptoms, Baek and Yoon concluded that food insecurity and poor psychological health exacerbate each other in a vicious cycle.

## Our reanalyses

As mentioned above, the RI-CLPM is susceptible to spurious findings. Therefore, we set out to scrutinize the findings and conclusions by Baek and Yoon by fitting alternative models to data. We have used this methodology previously and shown that prospective within-individual effects between academic self-concept and academic achievement (Marsh et al., 2024; Núñez-Regueiro et al., 2025), between curiosity and creativity (Ma and Wei, 2023), and between executive functions and symptoms of psychiatric disorders (Halse et al., 2022) may have been spurious (Sorjonen et al., 2025a,b; Sorjonen and Melin, 2025a,b).

**Figure 1:**
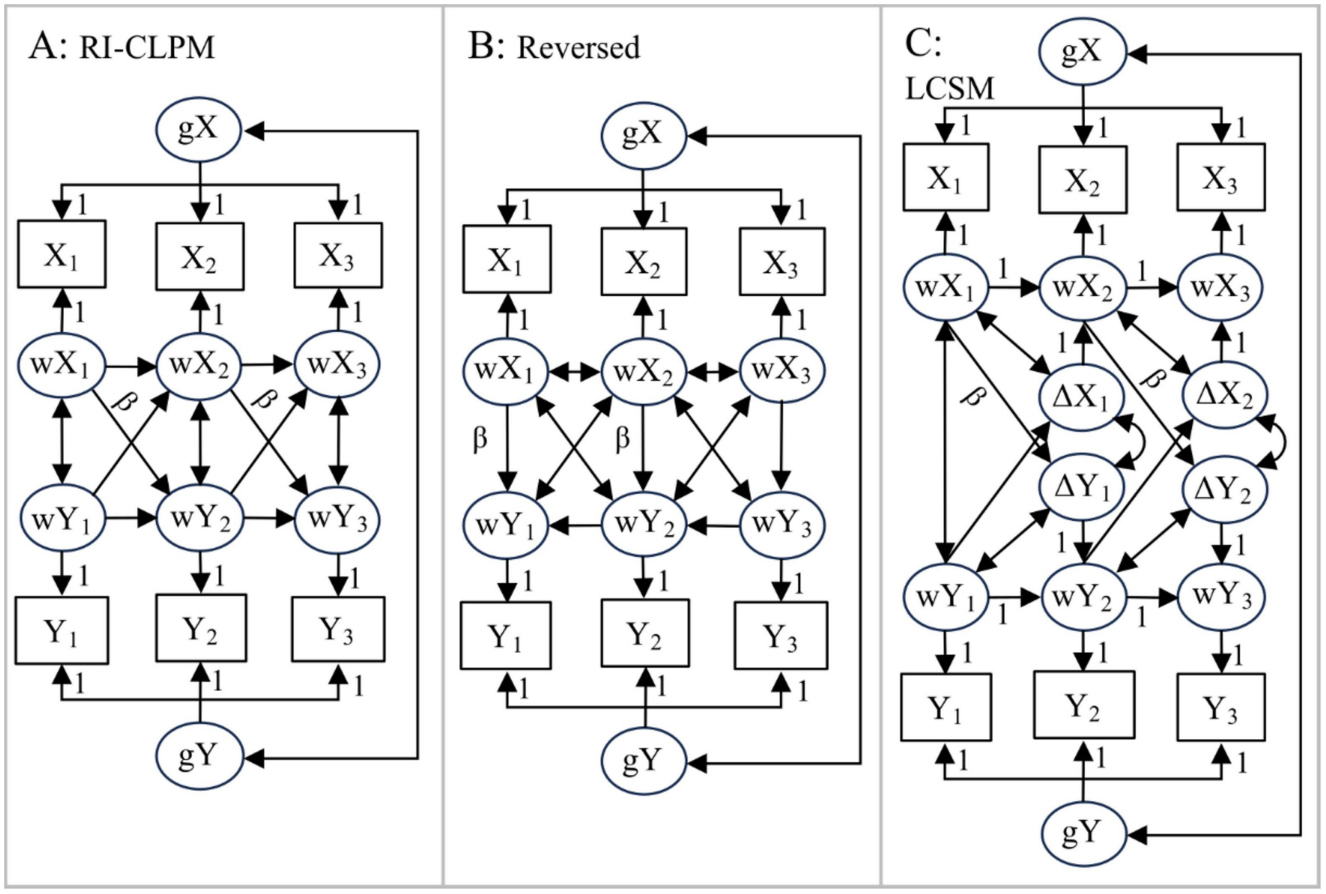
The analyzed models: **(A)** Original random-intercept cross-lagged panel model (RI-CLPM); **(B)** Reversed RI-CLPM; **(C)** Latent change score model (LCSM). *β* = focal effect; data contained eight waves of measurement, but for simplicity we only present the first three waves.

We fitted the three models in Figure 1 to data, separately for four combinations with food insecurity as the predictor and with either self-esteem or depressive symptoms as the outcome and vice versa. The three models were: (A) Original RI-CLPM; (B) Reversed RI-CLPM where within-individual X (e.g., food insecurity) at time T predicted within-individual Y (e.g., self-esteem) at time T while adjusting for within-individual Y at time T + 1. This model is in line with a recommendation by Campbell and Kenny (1999, see also Haufe et al., 2013) to use time-reversed analyses to identify statistical artifacts. With a genuine (i.e., non-spurious) prospective effect of X on Y, time-reversal should reverse the sign of the effect; (C) Latent change score model (LCSM, Ghisletta and McArdle, 2012; Kievit et al., 2018; McArdle, 2009) where latent change factors capture change in X and Y between T and T + 1. Then, X at time T predicts subsequent change in Y and vice versa. With a genuine prospective effect of X on Y, this effect should have the same sign as the corresponding effect in the RI-CLPM.

To elucidate the logic of time-reversed effects, we present an example in Table 1. Here, individuals with an X-score 1, 2, and 3 have experienced a decrease in the Y-score by 1, no change, and an increase by 1 from time 1 (Y_1_) to time 2 (Y_2_), respectively. This results in an effect of X on Y_2_ when adjusting for Y_1_ equal to *b* = 1 (i.e., among individuals with the same score on Y_1_, an increase in X by one is associated with an increase in Y_2_ by one) while the effect of X on Y_1_ when adjusting for Y_2_ is *b* = −1 (i.e., among individuals with the same score on Y_2_, an increase in X by one is associated with a decrease in Y_1_ by one). This latter negative effect indicates that a high score on X has counteracted a low score on Y_1_ and allowed individuals to reach the same score on Y_2_ as individuals with a higher score on Y_1_ but a lower score on X.

**Table 1:** Example of data where the effect of X on Y_2_ when adjusting for Y_1_ and of X on Y_1_ when adjusting for Y_2_ have opposite signs.

X	Y_1_	Y_2_
1	2	1
2	2	2
3	2	3
1	3	2
2	3	3
3	3	4
1	4	3
2	4	4
3	4	5

We conducted analyses with R 4.4.3 statistical software (R Core Team, 2026) using the lavaan package (Rosseel, 2012). The analytic script, containing the correlation matrix reported by Baek and Yoon (in their Table 2) used for the analyses reported here, is available at the Open Science Framework at https://osf.io/bvpgq/. As is possible in structural equation modelling, we fitted models to the correlation matrix rather than the Korean Welfare Panel Study (KoWePS) dataset used by Baek and Yoon. We fitted the models to the correlation matrix reported by Baek and Yoon rather than to the original individual-level KoWePS data. Under the estimation settings used here, the reported correlation matrix and sample size are sufficient to reproduce the covariance-structure analyses conducted in the present comment. The OSF repository contains the correlation matrix and analytic script, allowing readers to reproduce the findings reported below. However, these materials do not permit independent verification of the original data preparation, variable construction, sample selection, or handling of missing data. Interested readers may, upon registration, access the KoWePS data at https://www.koweps.re.kr:442/eng/main.do.

**Table 2:** Model fit and focal effects between food insecurity, self-esteem, and depressive symptoms.

Row	X	Y	Model	*β* (95% CI)	CFI	TLI	RMSEA (90% CI)
1	FI	SE	RI-CLPM	−0.012 (−0.018; −0.006)	0.978	0.978	0.027 (0.026; 0.028)
2	FI	SE	Reversed	−0.048 (−0.054; −0.042)	0.983	0.981	0.025 (0.024; 0.027)
3	FI	SE	LCSM	0.027 (0.021; 0.034)	0.980	0.978	0.027 (0.026; 0.029)
4	SE	FI	RI-CLPM	−0.014 (−0.022; −0.006)	0.978	0.978	0.027 (0.026; 0.028)
5	SE	FI	Reversed	−0.063 (−0.071; −0.055)	0.983	0.981	0.025 (0.024; 0.027)
6	SE	FI	LCSM	0.035 (0.027; 0.043)	0.980	0.978	0.027 (0.026; 0.029)
7	FI	DS	RI-CLPM	0.015 (0.008; 0.021)	0.971	0.972	0.029 (0.028; 0.030)
8	FI	DS	Reversed	0.071 (0.065; 0.078)	0.977	0.975	0.027 (0.026; 0.029)
9	FI	DS	LCSM	−0.047 (−0.053; −0.040)	0.975	0.972	0.029 (0.027; 0.030)
10	DS	FI	RI-CLPM	0.008 (0.000; 0.015)	0.971	0.972	0.029 (0.028; 0.030)
11	DS	FI	Reversed	0.086 (0.078; 0.093)	0.977	0.975	0.027 (0.026; 0.029)
12	DS	FI	LCSM	−0.063 (−0.071; −0.054)	0.975	0.972	0.029 (0.027; 0.030)

FI, food insecurity; SE, self-esteem; DS, depressive symptoms; The three models are illustrated in Figure 1; β, focal effect (see Figure 1); All effects were statistically significant (*p* < 0.05).

The three models in Figure 1 had good fit for all four combinations of X and Y variables (CFI/TLI > 0.97 and RMSEA < 0.03, Table 2). Findings with the original RI-CLPM indicated, in line with Baek and Yoon, negative (i.e., decreasing) prospective effects between food insecurity and self-esteem (Table 2, rows 1 and 4) and increasing prospective effects between food insecurity and depressive symptoms (Table 2, rows 7 and 10).

However, the reversed models suggested contradictory increasing prospective effects between food insecurity and self-esteem (Table 2, rows 2 and 5) and decreasing prospective effects between food insecurity and depressive symptoms (Table 2, rows 8 and 11). For example (Table 2, row 2), among individuals with the same within-individual self-esteem at time T + 1 (e.g., 0), those with higher within-individual food insecurity at time T (e.g., 1) were predicted to have had lower within-individual self-esteem at time T (1 × −0.048 = −0.048) and, hence, to have experienced a more positive change in self-esteem between T and T + 1 (0—(−0.048) = 0.048) compared to those with the same within-individual self-esteem at T + 1 but lower food insecurity at T (e.g., −1, −1 × −0.048 = 0.048, and 0–0.048 = −0.048). Here, the negative effect suggested that high food insecurity at T may have counteracted low self-esteem at T and allowed individuals to reach the same self-esteem at T + 1 as those with lower food insecurity but higher self-esteem at T. Likewise, a negative effect suggested that low initial self-esteem may have counteracted high initial food insecurity (Table 2, row 5), and positive effects suggested that high initial food insecurity may have counteracted high initial depressive symptoms (Table 2, row 8) and vice versa (Table 2, row 11).

Moreover, the LCSMs also suggested, contrary to the RI-CLPM, increasing prospective effects between food insecurity and self-esteem (Table 2, rows 3 and 6) and decreasing prospective effects between food insecurity and depressive symptoms (Table 2, rows 9 and 12).

## Alternative model

**Figure 2:**
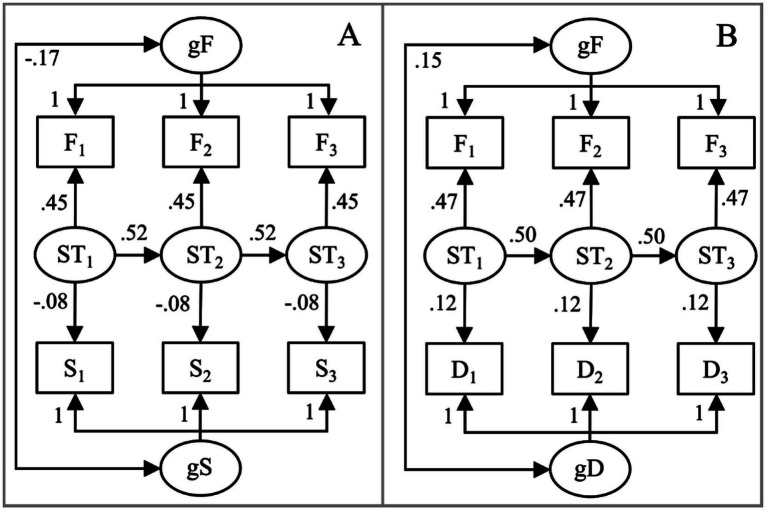
The model of spurious longitudinal associations (MoSLA) fitted to data on **(A)** food insecurity (F) and self-esteem (S); **(B)** food insecurity and depressive symptoms (D). Unstandardized estimates (although the manifest variables were standardized) (all *p* < 0.001). The model had good fit (*χ*^2^ = 2,581, *DF* = 130, *p* < 0.001, CFI = 0.957, TLI = 0.960, RMSEA = 0.037 [90% CI: 0.036; 0.038] in panel A and *χ*^2^ = 2,606, DF = 130, *p* < 0.001, CFI = 0.950, TLI = 0.954, RMSEA = 0.037 [90% CI: 0.036; 0.038] in panel B, respectively). gF, general food insecurity; gS, general self-esteem; gD, general depressive symptoms; ST, state factor; Data contained eight waves of measurement, but for simplicity we only present the first three waves.

We fitted the model of spurious longitudinal associations (MoSLA, Sorjonen and Melin, 2025a,b) to the data. In the MoSLA, longitudinal scores on two constructs are affected by trait factors as well as common autocorrelated state factors. Here, the state factors, affecting both self-rated food insecurity and self-esteem and depressive symptoms, could be things like mood, unemployment, psychiatric problems, etc. However, in the MoSLA, the longitudinal scores have no direct effects on one another, meaning that such effects identified by statistical models, e.g., the RI-CLPM, would be spurious. Here, the MoSLA had good fit (Figure 2), suggesting that the data analyzed by Baek and Yoon, and reanalyzed by us, may have been generated without prospective effects between food insecurity and self-esteem/depressive symptoms.

## Summary and concluding remarks

We set out to scrutinize findings and conclusions of prospective effects between food insecurity and self-esteem and depressive symptoms by Baek and Yoon. We fitted alternative models to data and found discrepant increasing and decreasing effects. Hence, the findings by Baek and Yoon may have been spurious and their conclusion, that food insecurity and poor psychological health exacerbate each other in a vicious cycle, may have been premature.

It is important for researchers to bear in mind that correlations, including effects in the random-intercept cross-lagged panel model, in observational (i.e., non-experimental) data may be spurious rather than due to genuine effects. We recommend researchers to, as we did here, fit alternative models to data and to base conclusions on a juxtaposition of findings. If findings from alternative models align, conclusions can be drawn with increased confidence. If, on the other hand and as in the present case, findings diverge, caution is advised and confident conclusions should be postponed.

This commentary is part of a research topic on promoting replicability. However, the present study is not an independent replication of Baek and Yoon, since it does not analyze data from a new sample. Rather, it examines the analytical robustness of the original conclusions by applying alternative plausible longitudinal models to the same reported correlation matrix. The fact that the direction and interpretation of the associations vary across defensible model specifications indicates that the substantive conclusions are model-dependent and therefore provides a less secure basis for expecting them to be reproduced consistently in independent samples. In general, for improved research planning and increased researcher responsibility, researchers should anticipate model uncertainty, consider alternative plausible longitudinal models or sensitivity analyses, and avoid drawing strong substantive conclusions when the findings depend on a single analytical specification.

### Limitations

We do not claim to have proven, once and for all, the null hypothesis that food insecurity, self-esteem, and depression have no genuine (i.e., non-spurious) prospective effects on one another. The more limited conclusion to be drawn is that the data used by Baek and Yoon, and reanalyzed by us, does not prove that the null hypothesis is false. Likewise, the good fit does not prove that the MoSLA was the true data generating model, as other models may also fit the data well. Rather, the good fit indicates that the MoSLA may have generated the data.

It would have been interesting to include measures of psychosocial disability, access to care and other resources, household composition, and qualitative or contextual evidence in the analyzed models. However, the required individual-level information is not available in the correlation matrix reported by Baek and Yoon and, moreover, such analyses would extend beyond the intended scope of this methodological comment.

The present report was intended as a methodological commentary, a warning against over interpreting associations in observational data, and a suggestion how researchers may increase rigor when analyzing longitudinal data. We used the study on food insecurity, self-esteem, and depression by Baek and Yoon as an example. However, this report was not intended as a comprehensive review of literature on food insecurity and mental health. Interested readers are recommended to engage with, for example, the following reviews and work that they cite: Abeldt (2024), Arenas et al. (2019), Myers (2020), Smith et al. (2024), and Teasdale et al. (2023).

## Data Availability

The analytic script, containing the correlation matrix reported by Baek and Yoon used for the analyses reported here, is available at the Open Science Framework at https://osf.io/bvpgq/.
